# Supplementary material for: Lipid Profiles of Human Milk and Infant Formulas: A Comparative Lipidomics Study
Source: Foods. 2023 Feb 1;12(3):600. doi: 10.3390/foods12030600 (PMC9914114; doi:10.3390/foods12030600)
Supplement: Supplementary file 1 [file foods-12-00600-s001.zip › foods-2113076-supplementary.pdf]

Table S1. Detailed information about the sources of fat in IFs

Brand	Age Rang Target (Months)	Fat Source	LC-PUFAs	OPO addition
IF1	0~6	Raw bovine milk, sunflower oil, coconut oil, flax oil, walnut oil	DHA, ARA	yes
IF2	0~6	skimmed milk, sunflower oil, soybean oil, coconut oil	DHA, ARA	no

Table S2. The content of lipid in positive ion mode

Adduct ion name	Average Mz	Average Rt(min)	Metabolite name	HM	IF1	IF2
[M+NH4]+	1070.98889	12.191	TG 66:3|TG 18:1_24:1_24:1	0.0004±0.0005	0.0006±0.0001	0.0005±0.0000
[M+NH4]+	1032.88501	11.207	TG 64:8|TG 24:0_20:4_20:4	0.0004±0.0003	0.0083±0.0003	0.0072±0.0002
[M+NH4]+	1042.96814	12.002	TG 64:3|TG 18:1_22:1_24:1	0.0030±0.0030	0.0011±0.0001	0.0009±0.0004
[M+NH4]+	1004.85132	10.968	TG 62:8|TG 22:0_20:4_20:4	0.0008±0.0005	0.0051±0.0001	0.0043±0.0002
[M+NH4]+	1008.88934	11.287	TG 62:6|TG 24:0_18:2_20:4	0.0041±0.0020	0.0034±0.0002	0.0054±0.0003
[M+NH4]+	1010.90033	11.457	TG 62:5|TG 24:0_18:1_20:4	0.0082±0.0023	0.0050±0.0007	0.0068±0.0003
[M+NH4]+	1014.9342	11.804	TG 62:3|TG 26:0_18:1_18:2	0.0222±0.0121	0.0072±0.0006	0.0091±0.0003
[M+NH4]+	1016.94958	12.004	TG 62:2|TG 26:0_18:1_18:1	0.0156±0.0101	0.0109±0.0014	0.0364±0.0009
[M+NH4]+	994.78705	9.76	TG 62:13|TG 18:1_22:6_22:6	0.0017±0.0008	0.0017±0.0000	0.0106±0.0002
[M+NH4]+	1002.93921	11.904	TG 61:2|TG 25:0_18:1_18:1	0.0042±0.0015	0.0055±0.0009	0.0232±0.0030
[M+NH4]+	984.88379	11.401	TG 60:4|TG 18:1_24:1_18:2	0.0523±0.0220	0.0645±0.0004	0.0554±0.0002
[M+NH4]+	986.9024	11.603	TG 60:3|TG 24:0_18:1_18:2	0.1230±0.0582	0.0524±0.0015	0.0611±0.0015
[M+NH4]+	988.91876	11.783	TG 60:2|TG 24:0_18:1_18:1	0.1201±0.0668	0.0776±0.0011	0.5590±0.0309
[M+NH4]+	988.92389	11.846	TG 60:2|TG 16:0_18:1_26:1	0.1201±0.0668	0.0776±0.0011	0.5590±0.0309
[M+NH4]+	966.74939	9.323	TG 60:13|TG 16:1_22:6_22:6	0.0009±0.0002	0.0027±0.0004	0.0154±0.0006
[M+NH4]+	972.79266	10.112	TG 60:10|TG 18:1_20:3_22:6	0.0098±0.0011	0.0079±0.0002	0.0038±0.0005
[M+NH4]+	972.88757	11.485	TG 59:3|TG 23:0_18:1_18:2	0.0066±0.0027	0.0101±0.0001	0.0170±0.0018
[M+NH4]+	974.90082	11.701	TG 59:2|TG 23:0_18:1_18:1	0.0156±0.0038	0.0167±0.0002	0.0703±0.0027
[M+NH4]+	976.89374	11.646	TG 59:1|TG 18:0_23:0_18:1	0.0023±0.0019	0.0074±0.0065	0.0086±0.0007
[M+NH4]+	946.78326	10.074	TG 58:9|TG 18:1_18:2_22:6	0.1564±0.0259	0.0156±0.0003	0.0225±0.0016
[M+NH4]+	948.79401	10.391	TG 58:8|TG 18:1_18:1_22:6	0.1036±0.0180	0.0466±0.0012	0.0527±0.0019
[M+NH4]+	950.81219	10.519	TG 58:7|TG 18:1_18:2_22:4	0.0871±0.0174	0.0060±0.0056	0.0158±0.0012

[M+NH4]+	950.81403	10.132	TG 58:7|TG 18:1_18:1_22:5	0.0086±0.0035	0.0014±0.0001	0.0033±0.0001
[M+NH4]+	952.83337	10.705	TG 58:6|TG 18:1_18:1_22:4	0.0559±0.0054	0.0025±0.0001	0.0034±0.0002
[M+NH4]+	956.85437	11.166	TG 58:4|TG 18:1_22:1_18:2	0.0893±0.0325	0.1106±0.0054	0.0899±0.0014
[M+NH4]+	958.87567	11.367	TG 58:3|TG 22:0_18:1_18:2	0.2365±0.0963	0.1278±0.0036	0.1855±0.0006
[M+NH4]+	960.888	11.591	TG 58:2|TG 16:0_18:1_24:1	0.3937±0.1779	0.2227±0.0011	1.3702±0.0336
[M+NH4]+	940.73474	9.261	TG 58:12|TG 14:0_22:6_22:6	0.0008±0.0009	0.0030±0.0001	0.0947±0.0040
[M+NH4]+	944.76422	9.756	TG 58:10|TG 18:2_18:2_22:6	0.0733±0.0070	0.0225±0.0002	0.0334±0.0026
[M+NH4]+	962.90796	11.814	TG 58:1|TG 16:0_24:0_18:1	0.2340±0.1748	0.1201±0.0062	0.1148±0.0003
[M+NH4]+	964.91956	12.021	TG 58:0|TG 16:0_18:0_24:0	0.0146±0.0112	0.0089±0.0001	0.0079±0.0111
[M+NH4]+	946.87213	11.478	TG 57:2|TG 21:0_18:1_18:1	0.0260±0.0043	0.0152±0.0008	0.0305±0.0002
[M+NH4]+	920.76648	9.883	TG 56:8|TG 18:2_18:2_20:4	0.1067±0.0948	0.0018±0.0002	0.0097±0.0002
[M+NH4]+	920.7688	10.062	TG 56:8|TG 16:0_18:2_22:6	0.1377±0.0642	0.0316±0.0007	0.0682±0.0029
[M+NH4]+	922.78931	10.174	TG 56:7|TG 18:1_18:2_20:4	0.2171±0.0525	0.0183±0.0009	0.0311±0.0007
[M+NH4]+	922.78314	10.36	TG 56:7|TG 16:0_18:1_22:6	0.1019±0.0210	0.0000±0.0000	0.0150±0.0034
[M+NH4]+	928.82672	10.929	TG 56:4|TG 18:1_20:1_18:2	0.1606±0.0226	0.0295±0.0026	0.0358±0.0009
[M+NH4]+	930.84161	11.128	TG 56:3|TG 18:1_18:1_20:1	0.2926±0.1029	0.0974±0.0060	0.2710±0.0107
[M+NH4]+	932.85724	11.362	TG 56:2|TG 16:0_18:1_22:1	0.4611±0.1610	0.2868±0.0017	0.5539±0.0132
[M+NH4]+	934.86829	11.312	TG 56:1|TG 18:0_20:0_18:1	0.0554±0.0181	0.0322±0.0035	0.0742±0.0133
[M+NH4]+	936.8847	11.846	TG 56:0|TG 16:0_18:0_22:0	0.0291±0.0205	0.0236±0.0006	0.0099±0.0112
[M+NH4]+	916.82739	10.994	TG 55:3|TG 18:1_18:1_19:1	0.0229±0.0104	0.0051±0.0004	0.0188±0.0009
[M+NH4]+	920.85199	11.469	TG 55:1|TG 16:0_21:0_18:1	0.0290±0.0104	0.0225±0.0000	0.0079±0.0018
[M+NH4]+	890.71875	9.077	TG 54:9|TG 18:3_18:3_18:3	0.0024±0.0042	0.7157±0.0143	0.0345±0.0023
[M+NH4]+	892.73346	9.376	TG 54:8|TG 18:2_18:3_18:3	0.0307±0.0126	0.6350±0.0179	0.4132±0.0156
[M+NH4]+	894.75262	11.412	TG 54:7|TG 18:2_18:2_18:3	0.0003±0.0001	0.0012±0.0001	0.0028±0.0005
[M+NH4]+	896.76202	11.22	TG 54:6|TG 18:2_18:2_18:2	0.0017±0.0003	0.0161±0.0022	0.0215±0.0036
[M+NH4]+	896.76343	10.025	TG 54:6|TG 18:1_18:2_18:3	1.8150±0.7105	4.1889±0.2828	4.5356±0.1524

[M+NH4]+	898.7749	9.963	TG 54:5|TG 18:1_18:2_18:2	0.2194±0.0745	0.4417±0.0669	0.5573±0.0516
[M+NH4]+	900.79388	10.612	TG 54:4|TG 18:1_18:1_18:2	2.5596±0.7349	1.9793±0.0594	3.2902±0.1497
[M+NH4]+	902.81189	10.874	TG 54:3|TG 18:1_18:1_18:1	1.8509±0.4736	1.0006±0.0185	4.0517±0.8521
[M+NH4]+	902.79681	10.616	TG 54:3|TG 18:0_18:1_18:2	0.2317±0.0909	0.1424±0.0102	0.2404±0.0351
[M+NH4]+	904.82849	11.112	TG 54:2|TG 18:0_18:1_18:1	1.4544±0.3674	1.2422±0.0510	2.7414±0.1118
[M+NH4]+	888.70465	9.186	TG 54:10|TG 12:0_20:4_22:6	0.0026±0.0007	0.0026±0.0001	0.0018±0.0002
[M+NH4]+	906.83887	11.362	TG 54:1|TG 16:0_20:0_18:1	0.6088±0.2006	0.6161±0.0025	0.2731±0.0148
[M+NH4]+	908.85199	11.593	TG 54:0|TG 16:0_18:0_20:0	0.0569±0.0321	0.0649±0.0017	0.0007±0.0007
[M+NH4]+	884.76935	10.133	TG 53:5|TG 17:1_18:2_18:2	0.0357±0.0202	0.0120±0.0007	0.0275±0.0001
[M+NH4]+	886.78345	10.421	TG 53:4|TG 17:1_18:1_18:2	0.0973±0.0627	0.0263±0.0012	0.0472±0.0017
[M+NH4]+	888.79114	10.653	TG 53:3|TG 17:1_18:1_18:1	0.1417±0.1072	0.0321±0.0013	0.0484±0.0003
[M+NH4]+	888.7962	10.759	TG 53:3|TG 17:0_18:1_18:2	0.1418±0.1073	0.0327±0.0013	0.0490±0.0003
[M+NH4]+	890.81073	10.971	TG 53:2|TG 17:0_18:1_18:1	0.1487±0.1240	0.0407±0.0016	0.0241±0.0039
[M+NH4]+	894.84723	11.491	TG 53:0|TG 17:0_18:0_18:0	0.0113±0.0088	0.0190±0.0004	0.0026±0.0004
[M+NH4]+	864.70587	9.311	TG 52:8|TG 12:0_18:2_22:6	0.0695±0.0177	0.0024±0.0011	0.0024±0.0004
[M+NH4]+	866.72211	9.429	TG 52:7|TG 12:0_18:1_22:6	0.0384±0.0030	0.0098±0.0073	0.0294±0.0005
[M+NH4]+	868.73615	9.602	TG 52:6|TG 16:0_18:3_18:3	0.3049±0.0167	0.3192±0.0032	0.1100±0.0031
[M+NH4]+	870.75006	10.033	TG 52:5|TG 16:0_18:2_18:3	1.2011±0.1584	0.2996±0.0171	0.6189±0.0249
[M+NH4]+	872.76819	10.298	TG 52:4|TG 16:0_18:2_18:2	3.1944±0.6917	2.2727±0.0749	2.3932±0.0857
[M+NH4]+	874.78394	10.574	TG 52:3|TG 16:0_18:1_18:2	4.7941±0.8689	2.5761±0.0007	1.7841±0.1245
[M+NH4]+	876.79785	10.856	TG 52:2|TG 16:0_18:1_18:1	5.4587±0.8518	2.5908±1.0071	0.9430±0.0202
[M+NH4]+	878.8103	11.106	TG 52:1|TG 16:0_18:0_18:1	3.1242±0.9515	1.7214±0.2313	0.3442±0.0052
[M+NH4]+	880.82269	11.223	TG 52:0|TG 16:0_18:0_18:0	0.2434±0.0803	0.2709±0.0025	0.0415±0.0032
[M+NH4]+	854.71497	9.527	TG 51:6|TG 15:1_18:2_18:3	0.0014±0.0003	0.0032±0.0000	0.0081±0.0004
[M+NH4]+	860.76184	10.432	TG 51:3|TG 15:0_18:1_18:2	0.1280±0.0850	0.0288±0.0003	0.0192±0.0007
[M+NH4]+	862.78461	10.711	TG 51:2|TG 16:0_17:1_18:1	0.1665±0.1399	0.0600±0.0015	0.0237±0.0007

[M+NH4]+	864.79462	10.985	TG 51:1|TG 16:0_17:0_18:1	0.1333±0.1262	0.0589±0.0030	0.0061±0.0001
[M+NH4]+	836.66974	8.883	TG 50:8|TG 10:0_18:2_22:6	0.0553±0.0132	0.0003±0.0002	0.0008±0.0005
[M+NH4]+	838.68903	9.24	TG 50:7|TG 10:0_18:1_22:6	0.1306±0.0423	0.0017±0.0006	0.0288±0.0001
[M+NH4]+	840.70416	9.629	TG 50:6|TG 14:0_14:0_22:6	0.1153±0.0340	0.0062±0.0087	0.1253±0.0055
[M+NH4]+	840.70673	9.39	TG 50:6|TG 12:0_18:2_20:4	0.1621±0.0222	0.0123±0.0000	0.1253±0.0055
[M+NH4]+	842.72113	9.679	TG 50:5|TG 14:0_18:2_18:3	0.5753±0.0986	0.0323±0.0008	0.0359±0.0013
[M+NH4]+	844.7345	9.84	TG 50:4|TG 14:0_18:2_18:2	0.0689±0.0290	0.0623±0.0001	0.0517±0.0037
[M+NH4]+	846.7511	10.242	TG 50:3|TG 14:0_18:1_18:2	1.6192±0.0541	0.3114±0.0043	0.1126±0.0034
[M+NH4]+	848.76331	11.887	TG 50:2|TG 16:0_16:1_18:1	0.0172±0.0122	0.0233±0.0165	0.0046±0.0005
[M+NH4]+	848.7666	10.531	TG 50:2|TG 16:0_16:0_18:2	1.8776±0.2412	0.9567±0.0039	0.4403±0.0265
[M+NH4]+	850.77557	10.809	TG 50:1|TG 16:0_16:0_18:1	1.1397±0.0859	1.5677±0.0534	0.0872±0.0231
[M+NH4]+	852.79449	11.097	TG 50:0|TG 16:0_16:0_18:0	0.1684±0.0552	0.3288±0.0329	0.0377±0.0001
[M+NH4]+	832.73279	10.139	TG 49:3|TG 13:0_18:1_18:2	0.0503±0.0261	0.0117±0.0007	0.0041±0.0002
[M+NH4]+	838.78546	11.545	TG 49:0|TG 14:0_17:0_18:0	0.0014±0.0017	0.0011±0.0001	0.0001±0.0000
[M+NH4]+	812.67303	8.999	TG 48:6|TG 12:0_18:3_18:3	0.0947±0.0346	0.0036±0.0003	0.1548±0.0041
[M+NH4]+	812.67773	9.219	TG 48:6|TG 12:0_14:0_22:6	0.1275±0.0487	0.0016±0.0000	0.1548±0.0041
[M+NH4]+	814.69165	9.298	TG 48:5|TG 12:0_18:2_18:3	0.6623±0.2890	0.0190±0.0006	0.0239±0.0010
[M+NH4]+	816.70697	9.593	TG 48:4|TG 12:0_18:2_18:2	2.5167±0.5138	0.0659±0.0003	0.0515±0.0012
[M+NH4]+	818.72186	9.91	TG 48:3|TG 12:0_18:1_18:2	3.0767±0.3213	0.1447±0.0038	0.1184±0.0008
[M+NH4]+	820.73389	10.04	TG 48:2|TG 14:0_16:0_18:2	0.2689±0.0546	0.4083±0.0048	0.2397±0.0077
[M+NH4]+	820.73395	10.222	TG 48:2|TG 12:0_18:1_18:1	2.1705±0.1865	0.4071±0.0048	0.2385±0.0077
[M+NH4]+	822.74963	10.508	TG 48:1|TG 14:0_16:0_18:1	0.7532±0.2134	0.5620±0.0028	0.1974±0.0076
[M+NH4]+	824.76025	10.785	TG 48:0|TG 14:0_16:0_18:0	0.0923±0.0566	0.1405±0.0059	0.0099±0.0005
[M+NH4]+	804.70593	9.731	TG 47:3|TG 12:0_17:1_18:2	0.0630±0.0213	0.0125±0.0008	0.0015±0.0008
[M+NH4]+	806.71887	10.085	TG 47:2|TG 12:0_17:0_18:2	0.0879±0.0484	0.0293±0.0024	0.0019±0.0001
[M+NH4]+	808.73608	10.395	TG 47:1|TG 12:0_17:0_18:1	0.0554±0.0384	0.0585±0.0029	0.0043±0.0005

[M+NH4]+	784.64471	8.763	TG 46:6|TG 12:0_12:0_22:6	0.0903±0.0393	0.0012±0.0003	0.0850±0.0011
[M+NH4]+	786.66022	8.864	TG 46:5|TG 10:0_18:2_18:3	0.3497±0.1259	0.0117±0.0062	0.0117±0.0001
[M+NH4]+	788.67462	9.191	TG 46:4|TG 10:0_18:2_18:2	1.4938±0.1703	0.0556±0.0002	0.0170±0.0006
[M+NH4]+	790.69177	9.551	TG 46:3|TG 10:0_18:1_18:2	2.9371±0.2035	0.1913±0.0011	0.0594±0.0003
[M+NH4]+	792.70703	9.892	TG 46:2|TG 12:0_16:0_18:2	3.2533±0.1729	0.4541±0.0108	0.2611±0.0068
[M+NH4]+	794.72113	10.192	TG 46:1|TG 12:0_16:0_18:1	2.4033±0.2691	0.7538±0.0004	0.5451±0.0164
[M+NH4]+	796.7312	10.497	TG 46:0|TG 12:0_16:0_18:0	0.1865±0.0735	0.2898±0.0050	0.1552±0.0078
[M+NH4]+	778.6911	9.743	TG 45:2|TG 10:0_17:1_18:1	0.1293±0.0637	0.0477±0.0002	0.0066±0.0001
[M+NH4]+	780.70264	10.06	TG 45:1|TG 12:0_15:0_18:1	0.0974±0.0624	0.0635±0.0005	0.0045±0.0009
[M+NH4]+	782.71472	10.115	TG 45:0|TG 14:0_15:0_16:0	0.0081±0.0086	0.0663±0.0016	0.0040±0.0003
[M+NH4]+	782.71393	10.382	TG 45:0|TG 12:0_16:0_17:0	0.0211±0.0154	0.0663±0.0016	0.0048±0.0002
[M+NH4]+	760.64618	8.734	TG 44:4|TG 8:0_18:2_18:2	0.4851±0.1582	0.0641±0.0004	0.0535±0.0011
[M+NH4]+	762.65692	9.156	TG 44:3|TG 8:0_18:1_18:2	1.5185±0.3812	0.1583±0.0020	0.1478±0.0008
[M+NH4]+	764.66919	10.421	TG 44:2|TG 10:0_16:1_18:1	0.0021±0.0021	0.0012±0.0002	0.0011±0.0001
[M+NH4]+	766.69025	9.842	TG 44:1|TG 12:0_14:0_18:1	3.5572±0.4677	1.3462±0.0232	1.0577±0.0303
[M+NH4]+	768.70453	10.176	TG 44:0|TG 12:0_14:0_18:0	0.5232±0.1496	0.7213±0.0155	0.8007±0.0368
[M+NH4]+	750.66058	9.298	TG 43:2|TG 10:0_15:0_18:2	0.0860±0.0350	0.0671±0.0003	0.0099±0.0003
[M+NH4]+	752.6778	9.65	TG 43:1|TG 10:0_15:0_18:1	0.1204±0.0683	0.1665±0.0023	0.0248±0.0015
[M+NH4]+	754.69073	10.028	TG 43:0|TG 10:0_16:0_17:0	0.0446±0.0325	0.0898±0.0034	0.0141±0.0111
[M+NH4]+	732.60736	8.286	TG 42:4|TG 8:0_16:1_18:3	0.2013±0.0854	0.1221±0.0040	0.0119±0.0006
[M+NH4]+	734.62665	8.713	TG 42:3|TG 12:0_12:0_18:3	1.1959±0.6135	0.2574±0.0112	0.0541±0.0002
[M+NH4]+	736.64526	9.095	TG 42:2|TG 12:0_12:0_18:2	3.6024±0.4026	0.7414±0.0138	0.4607±0.0003
[M+NH4]+	738.64893	9.101	TG 42:1|TG 12:0_12:0_18:1	0.3009±0.0242	0.0919±0.0003	0.0481±0.0021
[M+NH4]+	740.67163	9.824	TG 42:0|TG 12:0_14:0_16:0	1.1407±0.3935	1.9313±0.0400	2.9595±0.0334
[M+NH4]+	740.66766	9.438	TG 42:0|TG 10:0_14:0_18:0	0.3432±0.0293	0.1846±0.0101	0.2140±0.0037
[M+NH4]+	722.62292	8.861	TG 41:2|TG 11:0_12:0_18:2	0.0559±0.0136	0.0984±0.0040	0.0086±0.0004

[M+NH4]+	724.6438	9.546	TG 41:1|TG 8:0_16:0_17:1	0.0019±0.0010	0.0121±0.0016	0.0012±0.0005
[M+NH4]+	726.65625	9.636	TG 41:0|TG 10:0_15:0_16:0	0.0632±0.0391	0.2824±0.0114	0.0454±0.0019
[M+NH4]+	700.55322	7.047	TG 40:6|TG 8:0_10:0_22:6	0.0030±0.0025	0.0016±0.0002	0.0028±0.0003
[M+NH4]+	706.59753	8.228	TG 40:3|TG 10:0_12:0_18:3	0.7737±0.2376	0.8226±0.0349	0.0511±0.0003
[M+NH4]+	708.61023	8.635	TG 40:2|TG 10:0_12:0_18:2	3.2178±0.5013	1.8978±0.0634	0.3654±0.0026
[M+NH4]+	710.62701	9.034	TG 40:1|TG 10:0_12:0_18:1	3.7316±0.3366	3.4055±0.1242	1.1024±0.0099
[M+NH4]+	712.66296	9.195	TG 40:0|TG 12:0_14:0_14:0	0.3219±0.0261	0.2414±0.0663	6.0173±0.1427
[M+NH4]+	712.64111	9.427	TG 40:0|TG 10:0_14:0_16:0	2.1090±0.6847	3.5383±0.0542	6.0164±0.1427
[M+NH4]+	712.63892	9.009	TG 40:0|TG 10:0_12:0_18:0	0.3214±0.0261	0.2410±0.0663	0.1877±0.0026
[M+NH4]+	694.60071	8.142	TG 39:2|TG 12:0_12:0_15:2	0.0154±0.0015	0.1620±0.0066	0.0045±0.0002
[M+NH4]+	696.60999	9.213	TG 39:1|TG 12:0_13:0_14:1	0.0236±0.0400	0.0251±0.0000	0.0017±0.0002
[M+NH4]+	698.62543	9.231	TG 39:0|TG 10:0_12:0_17:0	0.0637±0.0333	0.4747±0.0123	0.0917±0.0049
[M+NH4]+	680.5777	8.134	TG 38:2|TG 8:0_12:0_18:2	0.9377±0.2398	2.3388±0.0780	0.4785±0.0046
[M+NH4]+	682.59497	8.583	TG 38:1|TG 8:0_12:0_18:1	2.0911±0.4275	5.5042±0.1393	2.1458±0.0716
[M+NH4]+	684.60382	8.569	TG 38:0|TG 8:0_12:0_18:0	0.2052±0.0131	0.4660±0.0055	0.2931±0.0089
[M+NH4]+	684.61243	8.98	TG 38:0|TG 12:0_12:0_14:0	3.0385±0.9206	6.1169±0.1069	8.4432±0.0886
[M+NH4]+	670.59552	8.776	TG 37:0|TG 10:0_12:0_15:0	0.0548±0.0247	0.9240±0.0159	0.1636±0.0056
[M+NH4]+	652.54871	7.562	TG 36:2|TG 8:0_10:0_18:2	0.1852±0.0566	0.8270±0.0237	0.1459±0.0039
[M+NH4]+	654.56427	8.082	TG 36:1|TG 8:0_10:0_18:1	0.4849±0.1534	3.7052±0.1675	0.6192±0.0001
[M+NH4]+	642.56256	7.997	TG 35:0|TG 11:0_11:0_13:0	0.0242±0.0043	0.0468±0.0021	0.0365±0.0001
[M+NH4]+	628.53949	7.422	TG 34:0|TG 8:0_8:0_18:0	0.0373±0.0095	0.2450±0.0124	0.1555±0.0007
[M+NH4]+	628.54773	7.958	TG 34:0|TG 10:0_12:0_12:0	1.7741±1.0714	8.0057±0.3988	10.9354±0.0682
[M+NH4]+	598.50238	6.818	TG 32:1|TG 8:0_10:0_14:1	0.0301±0.0058	0.7553±0.0104	0.0606±0.0018
[M+NH4]+	600.51605	7.357	TG 32:0|TG 10:0_10:0_12:0	0.4807±0.2653	6.8938±0.2777	10.9334±0.0802
[M+NH4]+	570.47144	6.148	TG 30:1|TG 8:0_8:0_14:1	0.0040±0.0012	0.5920±0.0153	0.0461±0.0003
[M+NH4]+	572.48804	5.986	TG 30:0|TG 8:0_8:0_14:0	0.0033±0.0024	0.2073±0.0130	0.1951±0.0041

[M+NH4]+	572.48602	6.705	TG 30:0|TG 8:0_10:0_12:0	0.0849±0.0518	3.2761±0.0852	6.8058±0.0992
[M+NH4]+	544.4549	5.859	TG 28:0|TG 8:0_8:0_12:0	0.0038±0.0058	1.7618±0.0371	2.6877±0.0140
[M+NH4]+	544.45544	6.586	TG 28:0|TG 8:0_10:0_10:0	0.0000±0.0000	0.0031±0.0021	0.0041±0.0000
[M+NH4]+	516.42407	5.178	TG 26:0|TG 8:0_8:0_10:0	0.0024±0.0010	0.6971±0.0272	0.3013±0.0050
[M+H]+	813.68134	13.515	SM 42:2;2O|SM 18:1;2O/24:1	0.0001±0.0000	0.0000±0.0000	0.0000±0.0000
[M+H]+	815.69958	7.252	SM 42:1;2O|SM 18:1;2O/24:0	0.0001±0.0000	0.0000±0.0000	0.0000±0.0000
[M+H]+	787.60559	9.317	SM 41:8;2O|SM 20:3;2O/21:5	0.0001±0.0000	0.0000±0.0000	0.0000±0.0000
[M+H]+	801.68884	5.759	SM 41:1;2O|SM 18:1;2O/23:0	0.0000±0.0000	0.0000±0.0000	0.0000±0.0000
[M+H]+	785.65125	7.761	SM 40:2;2O|SM 16:1;2O/24:1	0.0000±0.0000	0.0000±0.0000	0.0000±0.0000
[M+H]+	761.64697	8.838	SM 38:0;2O|SM 16:0;2O/22:0	0.0007±0.0001	0.0001±0.0000	0.0001±0.0000
[M+NH4]+	790.53857	9.575	PG 36:3|PG 18:1_18:2	0.0000±0.0000	0.0000±0.0000	0.0000±0.0000
[M+NH4]+	792.57648	9.888	PG 36:2|PG 18:1_18:1	0.0000±0.0000	0.0000±0.0000	0.0000±0.0000
[M+NH4]+	762.53955	9.203	PG 34:3|PG 14:0_20:3	0.0000±0.0000	0.0000±0.0000	0.0000±0.0000
[M+NH4]+	764.54163	4.024	PG 34:2|PG 16:0_18:2	0.0000±0.0000	0.0000±0.0000	0.0001±0.0000
[M+NH4]+	768.59912	10.114	PG 34:0|PG 16:0_18:0	0.0000±0.0000	0.0000±0.0000	0.0000±0.0000
[M+NH4]+	736.49664	9.055	PG 32:2|PG 14:0_18:2	0.0000±0.0000	0.0000±0.0000	0.0000±0.0000
[M+NH4]+	740.53711	9.806	PG 32:0|PG 16:0_16:0	0.0000±0.0000	0.0000±0.0000	0.0000±0.0000
[M+NH4]+	726.53418	9.564	PG 31:0|PG 15:0_16:0	0.0000±0.0000	0.0000±0.0000	0.0000±0.0000
[M+NH4]+	706.46613	8.258	PG 30:3|PG 12:0_18:3	0.0000±0.0000	0.0000±0.0000	0.0000±0.0000
[M+NH4]+	712.51904	9.42	PG 30:0|PG 14:0_16:0	0.0000±0.0000	0.0000±0.0000	0.0000±0.0000
[M+NH4]+	682.43896	8.555	PG 28:1|PG 12:0_16:1	0.0000±0.0000	0.0000±0.0000	0.0000±0.0000
[M+NH4]+	684.49878	8.961	PG 28:0|PG 14:0_14:0	0.0001±0.0000	0.0001±0.0000	0.0001±0.0000
[M+NH4]+	628.44183	7.932	PG 24:0|PG 12:0_12:0	0.0000±0.0000	0.0001±0.0000	0.0001±0.0000
[M+NH4]+	572.36768	6.658	PG 20:0|PG 8:0_12:0	0.0000±0.0000	0.0000±0.0000	0.0000±0.0000
[M+NH4]+	544.31366	5.871	PG 18:0|PG 6:0_12:0	0.0000±0.0000	0.0000±0.0000	0.0000±0.0000
[M+H]+	772.52325	5.754	PE P-40:8|PE P-18:2_22:6	0.0000±0.0000	0.0000±0.0000	0.0000±0.0000

[M+H]+	774.53937	6.382	PE P-40:7|PE P-18:1_22:6	0.0000±0.0000	0.0000±0.0000	0.0000±0.0000
[M+H]+	776.55804	6.636	PE P-40:6|PE P-18:1_22:5	0.0000±0.0000	0.0000±0.0000	0.0000±0.0000
[M+H]+	776.55695	7.065	PE P-40:6|PE P-18:0_22:6	0.0001±0.0000	0.0000±0.0000	0.0000±0.0000
[M+H]+	778.58167	7.155	PE P-40:5|PE P-18:1_22:4	0.0000±0.0000	0.0000±0.0000	0.0000±0.0000
[M+H]+	778.57574	7.309	PE P-40:5|PE P-18:0_22:5	0.0001±0.0000	0.0000±0.0000	0.0000±0.0000
[M+H]+	780.58197	7.818	PE P-40:4|PE P-18:0_22:4	0.0001±0.0001	0.0000±0.0000	0.0000±0.0000
[M+H]+	748.52667	5.974	PE P-38:6|PE P-18:2_20:4	0.0000±0.0000	0.0000±0.0000	0.0000±0.0000
[M+H]+	748.5235	6.318	PE P-38:6|PE P-16:0_22:6	0.0001±0.0000	0.0000±0.0000	0.0000±0.0000
[M+H]+	750.54156	6.598	PE P-38:5|PE P-18:1_20:4	0.0002±0.0001	0.0000±0.0000	0.0000±0.0000
[M+H]+	752.55939	7.298	PE P-38:4|PE P-18:0_20:4	0.0004±0.0002	0.0000±0.0000	0.0000±0.0000
[M+H]+	752.5592	7.132	PE P-38:4|PE P-16:0_22:4	0.0002±0.0001	0.0000±0.0000	0.0000±0.0000
[M+H]+	738.5332	6.912	PE P-37:4|PE P-17:0_20:4	0.0000±0.0000	0.0000±0.0000	0.0000±0.0000
[M+H]+	722.52045	5.97	PE P-36:5|PE P-16:0_20:5	0.0000±0.0000	0.0000±0.0000	0.0000±0.0000
[M+H]+	724.52765	6.214	PE P-36:4|PE P-18:2_18:2	0.0000±0.0000	0.0000±0.0000	0.0000±0.0000
[M+H]+	724.52771	6.549	PE P-36:4|PE P-16:0_20:4	0.0004±0.0001	0.0000±0.0000	0.0000±0.0000
[M+H]+	726.54315	6.825	PE P-36:3|PE P-18:1_18:2	0.0002±0.0001	0.0000±0.0000	0.0000±0.0000
[M+H]+	728.55591	7.508	PE P-36:2|PE P-18:0_18:2	0.0003±0.0001	0.0000±0.0000	0.0000±0.0000
[M+H]+	730.57214	8.08	PE P-36:1|PE P-16:0_20:1	0.0002±0.0001	0.0003±0.0001	0.0000±0.0000
[M+H]+	700.52667	6.727	PE P-34:2|PE P-16:0_18:2	0.0003±0.0001	0.0000±0.0000	0.0001±0.0000
[M+H]+	702.54218	7.32	PE P-34:1|PE P-16:0_18:1	0.0003±0.0001	0.0002±0.0000	0.0001±0.0001
[M+H]+	792.54944	6.676	PE 40:6|PE 16:1_24:5	0.0001±0.0001	0.0000±0.0000	0.0000±0.0000
[M+H]+	798.60339	7.768	PE 40:3|PE 22:1_18:2	0.0000±0.0000	0.0000±0.0000	0.0000±0.0000
[M+H]+	740.51813	6.146	PE 36:4|PE 16:0_20:4	0.0000±0.0000	0.0000±0.0000	0.0000±0.0000
[M+H]+	744.55042	7.133	PE 36:2|PE 18:0_18:2	0.0019±0.0012	0.0009±0.0012	0.0002±0.0001
[M+H]+	716.43225	6.305	PE 35:9|PE 17:4_18:5	0.0000±0.0000	0.0000±0.0000	0.0000±0.0000
[M+H]+	718.43781	6.961	PE 35:8|PE 17:3_18:5	0.0000±0.0000	0.0000±0.0000	0.0000±0.0000

[M+H]+	692.5163	6.818	PE 32:0|PE 16:0_16:0	0.0000±0.0000	0.0001±0.0000	0.0001±0.0000
[M+H]+	412.18964	1.103	PE 12:0|PE 6:0_6:0	0.0000±0.0000	0.0000±0.0000	0.0000±0.0000
[M+H]+	810.60486	8.713	PC 38:4|PC 18:0_20:4	0.0001±0.0000	0.0000±0.0000	0.0000±0.0000
[M+H]+	818.66315	7.856	PC 38:0|PC 19:0_19:0	0.0005±0.0003	0.0000±0.0000	0.0000±0.0000
[M+H]+	796.60223	5.43	PC 37:4|PC 17:0_20:4	0.0025±0.0017	0.0022±0.0030	0.0013±0.0006
[M+H]+	780.55017	6.06	PC 36:5|PC 18:2_18:3	0.0000±0.0000	0.0002±0.0001	0.0006±0.0001
[M+H]+	782.54309	6.875	PC 36:4|PC 18:2_18:2	0.0002±0.0001	0.0010±0.0003	0.0065±0.0011
[M+H]+	784.5791	8.346	PC 36:3|PC 18:1_18:2	0.0002±0.0002	0.0002±0.0000	0.0004±0.0001
[M+H]+	786.60065	9.482	PC 36:2|PC 18:0_18:2	0.0068±0.0028	0.0011±0.0010	0.0008±0.0002
[M+H]+	788.60925	10.74	PC 36:1|PC 18:0_18:1	0.0001±0.0001	0.0000±0.0000	0.0000±0.0000
[M+H]+	790.64154	5.428	PC 36:0|PC 17:0_19:0	0.0000±0.0000	0.0000±0.0000	0.0000±0.0000
[M+H]+	774.60144	10.121	PC 35:1|PC 16:0_19:1	0.0000±0.0000	0.0000±0.0000	0.0000±0.0000
[M+H]+	758.56891	7.937	PC 34:2|PC 16:0_18:2	0.0018±0.0009	0.0023±0.0006	0.0033±0.0006
[M+H]+	760.59137	9.069	PC 34:1|PC 16:0_18:1	0.0020±0.0012	0.0035±0.0019	0.0029±0.0006
[M+H]+	762.59625	11.634	PC 34:0|PC 16:0_18:0	0.0002±0.0002	0.0000±0.0000	0.0000±0.0000
[M+H]+	706.53949	7.77	PC 30:0|PC 14:0_16:0	0.0003±0.0001	0.0024±0.0017	0.0014±0.0001
[M+NH4]+	642.56097	8.744	MG 38:7	0.0002±0.0002	0.1100±0.1527	0.0015±0.0001
[M+NH4]+	610.48718	7.203	MG 36:9	0.0002±0.0002	0.0010±0.0003	0.0008±0.0001
[M+NH4]+	616.53668	7.691	MG 36:6	0.0006±0.0004	0.0331±0.0032	0.0123±0.0021
[M+NH4]+	612.59259	7.746	MG 35:1	0.0626±0.0225	0.0136±0.0005	0.0042±0.0005
[M+NH4]+	528.40234	5.752	MG 30:8	0.0002±0.0000	0.0000±0.0000	0.0001±0.0000
[M+NH4]+	530.4057	6.435	MG 30:7	0.0002±0.0002	0.0003±0.0002	0.0002±0.0003
[M+NH4]+	528.51276	5.742	MG 29:1	0.0152±0.0010	0.0000±0.0000	0.0000±0.0000
[M+NH4]+	398.3233	2.057	MG 20:3	0.0016±0.0005	0.0000±0.0000	0.0001±0.0000
[M+NH4]+	370.29529	1.466	MG 18:3	0.0027±0.0003	0.0039±0.0011	0.0025±0.0004
[M+NH4]+	372.30896	1.824	MG 18:2	0.0256±0.0189	0.0187±0.0031	0.0174±0.0021

[M+NH4]+	376.34229	3.038	MG 18:0	0.3002±0.0173	0.5114±0.0453	0.4946±0.0347
[M+NH4]+	348.31125	2.227	MG 16:0	0.1501±0.0738	0.3519±0.0333	0.3133±0.0503
[M+H]+	574.40778	2.4	LPC 22:3	0.0000±0.0000	0.0000±0.0000	0.0000±0.0000
[M+H]+	564.39685	2.428	LPC 21:1	0.0001±0.0001	0.0005±0.0001	0.0003±0.0001
[M+H]+	798.67279	8.064	HexCer 41:1;2O|HexCer 18:1;2O/23:0	0.0000±0.0000	0.0000±0.0000	0.0000±0.0000
[M+H]+	728.60516	6.601	HexCer 36:1;2O|HexCer 18:1;2O/18:0	0.0000±0.0000	0.0000±0.0000	0.0000±0.0000
[M+H]+	700.57275	5.81	HexCer 34:1;2O|HexCer 18:1;2O/16:0	0.0000±0.0000	0.0000±0.0000	0.0000±0.0000
[M+H]+	974.74701	8.051	Hex2Cer 42:1;2O|Hex2Cer 18:1;2O/24:0	0.0000±0.0000	0.0000±0.0000	0.0000±0.0000
[M+H]+	960.72235	7.715	Hex2Cer 41:1;2O|Hex2Cer 18:1;2O/23:0	0.0000±0.0000	0.0000±0.0000	0.0000±0.0000
[M+H]+	946.71777	7.496	Hex2Cer 40:1;2O|Hex2Cer 18:1;2O/22:0	0.0000±0.0000	0.0000±0.0000	0.0000±0.0000
[M+H]+	932.70166	7.16	Hex2Cer 39:1;2O|Hex2Cer 16:1;2O/23:0	0.0000±0.0000	0.0000±0.0000	0.0000±0.0000
[M+H]+	918.69092	6.889	Hex2Cer 38:1;2O|Hex2Cer 16:1;2O/22:0	0.0000±0.0000	0.0000±0.0000	0.0000±0.0000
[M+H]+	862.61743	5.534	Hex2Cer 34:1;2O|Hex2Cer 18:1;2O/16:0	0.0000±0.0000	0.0000±0.0000	0.0000±0.0000
[M+NH4]+	682.54059	6.327	DG 40:8|DG 18:2_22:6	0.0434±0.0082	0.0141±0.0010	0.0142±0.0000
[M+NH4]+	684.55548	6.952	DG 40:7|DG 18:1_22:6	0.0542±0.0047	0.0002±0.0000	0.0056±0.0010
[M+NH4]+	694.63141	8.854	DG 40:2|DG 18:1_22:1	0.0063±0.0026	0.0106±0.0002	0.0031±0.0002
[M+NH4]+	678.5871	8.172	DG 39:3|DG 21:1_18:2	0.0004±0.0003	0.0001±0.0001	0.0001±0.0000
[M+NH4]+	656.52423	6.115	DG 38:7|DG 18:3_20:4	0.0034±0.0009	0.0029±0.0001	0.0075±0.0004
[M+NH4]+	658.54193	6.682	DG 38:6|DG 18:2_20:4	0.0339±0.0126	0.0056±0.0001	0.0124±0.0005
[M+NH4]+	658.54077	7.032	DG 38:6|DG 16:0_22:6	0.0262±0.0020	0.0011±0.0010	0.0007±0.0002
[M+NH4]+	660.55878	7.171	DG 38:5|DG 18:1_20:4	0.0584±0.0141	0.0138±0.0001	0.0076±0.0006
[M+NH4]+	664.5899	7.853	DG 38:3|DG 20:1_18:2	0.0370±0.0119	0.0177±0.0005	0.0034±0.0004
[M+NH4]+	666.60126	8.321	DG 38:2|DG 18:1_20:1	0.0238±0.0088	0.0464±0.0002	0.0110±0.0012
[M+NH4]+	630.50775	5.832	DG 36:6|DG 18:3_18:3	0.0114±0.0062	0.0641±0.0026	0.0148±0.0007
[M+NH4]+	632.52557	6.321	DG 36:5|DG 18:2_18:3	0.0957±0.0249	0.1515±0.0077	0.1876±0.0070
[M+NH4]+	634.54138	6.766	DG 36:4|DG 18:2_18:2	0.4760±0.2385	1.4039±0.0060	0.8381±0.0459

[M+NH4]+	636.55615	7.351	DG 36:3|DG 18:1_18:2	1.1061±0.6645	2.1658±0.0138	0.3947±0.0301
[M+NH4]+	638.56055	7.293	DG 36:2|DG 18:1_18:1	0.1063±0.0490	0.1301±0.0059	0.0305±0.0007
[M+NH4]+	640.58606	8.304	DG 36:1|DG 18:0_18:1	0.0855±0.0205	0.3805±0.0066	0.0777±0.0041
[M+NH4]+	642.60217	8.789	DG 36:0|DG 18:0_18:0	0.1895±0.0474	0.0581±0.0013	0.0607±0.0006
[M+NH4]+	622.53656	7.017	DG 35:3|DG 17:1_18:2	0.0119±0.0037	0.0061±0.0008	0.0032±0.0005
[M+NH4]+	624.55444	7.519	DG 35:2|DG 17:0_18:2	0.0246±0.0121	0.0170±0.0011	0.0056±0.0005
[M+NH4]+	602.47821	5.408	DG 34:6|DG 12:0_22:6	0.0249±0.0066	0.0084±0.0001	0.0132±0.0001
[M+NH4]+	608.52344	6.716	DG 34:3|DG 16:1_18:2	0.2643±0.0406	0.2669±0.0020	0.0463±0.0024
[M+NH4]+	612.54718	7.272	DG 34:1|DG 18:0_16:1	0.1023±0.0410	0.0671±0.0043	0.0106±0.0135
[M+NH4]+	612.55365	7.739	DG 34:1|DG 16:0_18:1	1.4245±0.4540	3.2931±0.0993	0.2811±0.0232
[M+NH4]+	614.56042	7.724	DG 34:0|DG 16:0_18:0	0.0929±0.0274	0.1080±0.0041	0.0038±0.0011
[M+NH4]+	600.55341	8.04	DG 33:0|DG 16:0_17:0	0.0081±0.0050	0.0163±0.0005	0.0035±0.0010
[M+NH4]+	580.49194	6.05	DG 32:3|DG 14:0_18:3	0.0486±0.0235	0.0099±0.0005	0.0021±0.0008
[M+NH4]+	582.50751	6.554	DG 32:2|DG 14:0_18:2	0.2980±0.0642	0.0495±0.0019	0.0299±0.0023
[M+NH4]+	584.52417	7.116	DG 32:1|DG 14:0_18:1	0.4431±0.0569	0.2283±0.0152	0.0922±0.0053
[M+NH4]+	568.49255	6.22	DG 31:2|DG 13:0_18:2	0.0126±0.0143	0.0003±0.0001	0.0002±0.0001
[M+NH4]+	552.46216	5.231	DG 30:3|DG 12:0_18:3	0.0463±0.0320	0.0021±0.0000	0.0010±0.0001
[M+NH4]+	554.47632	5.805	DG 30:2|DG 12:0_18:2	0.4310±0.1060	0.0146±0.0011	0.0360±0.0019
[M+NH4]+	556.49414	6.424	DG 30:1|DG 12:0_18:1	0.5002±0.1206	0.0787±0.0026	0.2128±0.0070
[M+NH4]+	558.50751	7.064	DG 30:0|DG 14:0_16:0	0.1063±0.0095	0.2619±0.0141	0.1841±0.0021
[M+NH4]+	558.49847	6.445	DG 30:0|DG 12:0_18:0	0.0495±0.0035	0.0063±0.0085	0.0375±0.0009
[M+NH4]+	540.46338	5.465	DG 29:2|DG 11:0_18:2	0.0022±0.0009	0.0004±0.0001	0.0002±0.0001
[M+NH4]+	526.44708	4.993	DG 28:2|DG 10:0_18:2	0.1324±0.0251	0.0052±0.0001	0.0047±0.0000
[M+NH4]+	528.46075	5.677	DG 28:1|DG 10:0_18:1	0.2950±0.0296	0.0266±0.0010	0.0283±0.0014
[M+NH4]+	530.47827	6.365	DG 28:0|DG 12:0_16:0	0.2421±0.0199	0.1086±0.0074	0.3724±0.0116
[M+NH4]+	512.42969	4.58	DG 27:2|DG 9:0_18:2	0.0006±0.0001	0.0005±0.0003	0.0003±0.0000

[M+NH4]+	514.45264	5.597	DG 27:1|DG 12:0_15:1	0.0014±0.0005	0.0001±0.0000	0.0003±0.0001
[M+NH4]+	516.46295	6.046	DG 27:0|DG 12:0_15:0	0.0054±0.0023	0.0020±0.0026	0.0007±0.0006
[M+NH4]+	498.41061	4.717	DG 26:2|DG 8:0_18:2	0.0004±0.0003	0.0002±0.0001	0.0004±0.0002
[M+NH4]+	500.4332	5.203	DG 26:1|DG 10:0_16:1	0.0238±0.0311	0.0076±0.0106	0.0010±0.0001
[M+NH4]+	502.44559	5.578	DG 26:0|DG 12:0_14:0	0.2684±0.0420	0.0847±0.0027	0.6001±0.0027
[M+NH4]+	488.43015	5.174	DG 25:0|DG 10:0_15:0	0.0028±0.0014	0.0022±0.0003	0.0010±0.0009
[M+NH4]+	474.41327	4.711	DG 24:0|DG 12:0_12:0	0.1353±0.0462	0.0745±0.0036	0.6186±0.0194
[M+H]+	700.57904	6.77	CerP 40:2;2O|CerP 18:0;2O/22:2	0.0000±0.0000	0.0000±0.0000	0.0000±0.0000
[M+H]+	512.50043	7.37	Cer(d18:0/14:0)	0.0027±0.0011	0.0035±0.0004	0.0019±0.0001

Table S3 The content of lipid in negative ion mode

Adduct ion name	Average Mz	Average Rt(min)	Metabolite name	HM	IF1	IF2
[M+AcO-H]-	871.68781	3.401	SM 42:2;2O|SM 25:1;2O/17:1	0.4848±0.1003	0.0016±0.0016	0.0003±0.0004
[M+AcO-H]-	871.68134	12.982	SM 42:2;2O|SM 18:1;2O/24:1	1.0270±0.9140	0.0011±0.0007	0.0000±0.0000
[M+AcO-H]-	845.59735	9.387	SM 41:8;2O|SM 21:3;2O/20:5	1.1377±0.6065	0.0206±0.0026	0.0259±0.0042
[M+AcO-H]-	843.65887	11.012	SM 40:2;2O|SM 22:0;2O/18:2	0.2279±0.0534	0.0199±0.0023	0.0143±0.0034
[M+AcO-H]-	845.67133	3.383	SM 40:1;2O|SM 25:1;2O/15:0	0.3300±0.0654	0.0000±0.0000	0.0096±0.0010
[M+AcO-H]-	845.67084	14.735	SM 40:1;2O|SM 18:1;2O/22:0	0.8831±0.4904	0.0133±0.0015	0.0004±0.0001
[M+AcO-H]-	817.63861	10.788	SM 38:1;2O|SM 18:1;2O/20:0	1.4921±0.4334	0.0006±0.0009	0.0106±0.0057
[M+AcO-H]-	787.59314	7.813	SM 36:2;2O|SM 16:1;2O/20:1	0.1940±0.1356	0.0164±0.0006	0.0046±0.0033
[M+AcO-H]-	789.60913	9.075	SM 36:1;2O|SM 18:1;2O/18:0	5.7032±1.7099	0.1465±0.0086	0.0144±0.0023
[M+AcO-H]-	791.62769	9.541	SM 36:0;2O|SM 18:0;2O/18:0	0.3824±0.0912	0.0299±0.0101	0.0050±0.0036
[M+AcO-H]-	761.57788	7.569	SM 34:1;2O|SM 18:1;2O/16:0	1.5717±0.1599	0.4729±0.0676	0.3725±0.1830
[M-H]-	834.51825	4.759	PS 40:6|PS 18:0_22:6	0.1877±0.1103	0.0262±0.0191	0.0393±0.0344
[M-H]-	836.55414	5.239	PS 40:5|PS 18:0_22:5	0.0607±0.0440	0.0520±0.0501	0.0342±0.0278
[M-H]-	838.5498	5.599	PS 40:4|PS 18:0_22:4	0.0516±0.0434	0.0092±0.0005	0.0113±0.0015
[M-H]-	812.54639	5.258	PS 38:3|PS 18:0_20:3	0.1315±0.1325	0.5825±0.1098	0.3593±0.0818
[M-H]-	814.56079	5.844	PS 38:2|PS 18:0_20:2	0.0322±0.0268	0.0107±0.0043	0.0477±0.0197
[M-H]-	784.52002	4.397	PS 36:3|PS 18:1_18:2	0.0650±0.0642	0.0679±0.0246	0.2793±0.1104
[M-H]-	784.50769	4.539	PS 36:3|PS 18:0_18:3	0.0655±0.0636	0.0657±0.0276	0.2349±0.1731
[M-H]-	786.52887	5.767	PS 36:2|PS 18:1_18:1	0.1230±0.1066	0.0102±0.0027	0.0523±0.0087
[M-H]-	786.52429	5.064	PS 36:2|PS 18:0_18:2	1.5090±1.0926	0.1413±0.1780	0.9285±0.0864
[M-H]-	788.54456	5.499	PS 36:1|PS 18:0_18:1	0.5216±0.7344	0.3108±0.0852	0.9088±0.1236
[M-H]-	760.51343	4.728	PS 34:1|PS 16:0_18:1	0.0000±0.0000	0.0000±0.0000	0.2829±0.0658

[M-H]-	762.51575	5.629	PS 34:0|PS 16:0_18:0	0.0252±0.0120	0.0869±0.0452	0.1075±0.0205
[M-H]-	909.5473	4.403	PI 40:6|PI 18:0_22:6	0.0480±0.0152	0.0014±0.0009	0.0023±0.0009
[M-H]-	911.57123	4.9	PI 40:5|PI 18:0_22:5	0.0134±0.0045	0.0012±0.0016	0.0006±0.0009
[M-H]-	883.52521	3.952	PI 38:5|PI 18:1_20:4	0.3823±0.1008	0.3750±0.1708	0.0904±0.0341
[M-H]-	885.54193	4.233	PI 38:4|PI 18:1_20:3	0.1471±0.0518	0.2054±0.0286	0.0570±0.0462
[M-H]-	885.54657	4.608	PI 38:4|PI 18:0_20:4	1.4176±0.3211	0.7868±0.0972	0.1267±0.1748
[M-H]-	887.55963	4.918	PI 38:3|PI 18:0_20:3	0.8983±0.3649	0.7140±0.1201	0.1234±0.1456
[M-H]-	871.52698	4.222	PI 37:4|PI 17:0_20:4	0.0081±0.0039	0.0141±0.0047	0.0004±0.0006
[M-H]-	855.50775	2.776	PI 36:5|PI 18:2_18:3	0.0002±0.0004	0.1184±0.0039	0.4024±0.0320
[M-H]-	857.52155	3.28	PI 36:4|PI 18:2_18:2	0.0001±0.0002	0.6855±0.0836	2.4734±0.1620
[M-H]-	857.51196	3.841	PI 36:4|PI 16:0_20:4	0.0529±0.0225	0.0793±0.1064	0.0175±0.0067
[M-H]-	859.52386	4.014	PI 36:3|PI 18:1_18:2	0.5614±0.1107	0.6966±0.5165	1.6580±0.0036
[M-H]-	861.55078	4.539	PI 36:2|PI 18:1_18:1	1.8803±0.0963	3.6203±0.4107	3.3588±0.1161
[M-H]-	861.54681	4.725	PI 36:2|PI 18:0_18:2	1.8803±0.0963	3.6203±0.4107	3.3588±0.1161
[M-H]-	863.55865	5.287	PI 36:1|PI 18:0_18:1	0.5403±0.0974	1.2804±1.6010	1.2542±0.0705
[M-H]-	865.57501	5.888	PI 36:0|PI 18:0_18:0	0.0059±0.0044	0.0506±0.0138	0.0218±0.0129
[M-H]-	831.50659	3.288	PI 34:3|PI 16:0_18:3	0.0062±0.0069	0.9444±0.1908	1.7367±0.1802
[M-H]-	833.51483	3.97	PI 34:2|PI 16:0_18:2	0.2078±0.0437	4.2406±0.5672	13.2752±0.4234
[M-H]-	835.53156	4.538	PI 34:1|PI 16:0_18:1	0.4548±0.0971	1.4911±0.1839	2.7414±0.1609
[M-H]-	807.50427	3.774	PI 32:1|PI 14:0_18:1	0.0381±0.0140	0.2406±0.0447	1.0084±0.0303
[M-H]-	781.48712	3.729	PI 30:0|PI 12:0_18:0	0.0151±0.0113	0.1121±0.0218	0.0038±0.0020
[M-H]-	795.54407	9.067	PG 38:5|PG 18:1_20:4	0.0196±0.0046	0.0337±0.0203	0.0099±0.0037
[M-H]-	777.56451	6.086	PG 36:0|PG 17:0_19:0	0.2292±0.0878	0.2612±0.0484	0.0080±0.0056
[M-H]-	763.53772	5.766	PG 35:0|PG 17:0_18:0	0.1382±0.0227	0.1069±0.0121	0.0418±0.0087
[M-H]-	735.51538	5.056	PG 33:0|PG 16:0_17:0	0.0390±0.0111	0.0280±0.0345	0.0033±0.0026
[M-H]-	828.64948	9.428	PE 42:1|PE 24:0_18:1	0.0227±0.0068	0.0528±0.0129	0.0569±0.0012

[M-H]-	790.53558	6.685	PE 40:6|PE 18:0_22:6	0.8833±0.2594	0.0331±0.0227	0.0257±0.0176
[M-H]-	792.54974	7.202	PE 40:5|PE 18:0_22:5	0.5398±0.2734	0.0011±0.0015	0.0000±0.0000
[M-H]-	794.56445	7.424	PE 40:4|PE 18:0_22:4	0.5178±0.3071	0.0546±0.0508	0.0263±0.0247
[M-H]-	798.59479	8.331	PE 40:2|PE 18:1_22:1	0.3972±0.3322	0.1476±0.0138	0.2230±0.0021
[M-H]-	800.62225	7.18	PE 40:1|PE 22:0_18:1	0.0218±0.0027	0.1770±0.0205	0.1119±0.0256
[M-H]-	800.61578	8.942	PE 40:1|PE 18:0_22:1	0.5457±0.3216	0.1231±0.0126	0.0951±0.0209
[M-H]-	764.51862	6.217	PE 38:5|PE 18:1_20:4	0.3424±0.0587	0.1837±0.1627	0.1344±0.0314
[M-H]-	766.53076	6.495	PE 38:4|PE 18:2_20:2	0.1492±0.0441	0.3749±0.2646	0.2050±0.0514
[M-H]-	766.54175	6.574	PE 38:4|PE 18:1_20:3	0.1492±0.0441	0.3274±0.3317	0.2566±0.0216
[M-H]-	766.53247	6.905	PE 38:4|PE 18:0_20:4	1.8568±0.2241	0.2885±0.3874	0.2808±0.0122
[M-H]-	768.55249	7.268	PE 38:3|PE 18:0_20:3	1.7124±0.4769	0.2837±0.0282	0.2392±0.0023
[M-H]-	770.56415	9.128	PE 38:2|PE 18:1_20:1	0.2610±0.1212	0.0482±0.0375	0.0193±0.0107
[M-H]-	770.57074	7.779	PE 38:2|PE 18:0_20:2	1.2929±0.6368	0.1290±0.1065	0.2780±0.0096
[M-H]-	772.58032	8.331	PE 38:1|PE 18:0_20:1	2.3977±1.1989	0.1883±0.0088	0.0703±0.0546
[M-H]-	774.58911	8.316	PE 38:0|PE 18:0_20:0	0.2556±0.1151	0.0373±0.0118	0.0170±0.0073
[M-H]-	736.48962	4.974	PE 36:5|PE 18:2_18:3	0.0096±0.0088	0.0560±0.0031	1.0639±0.0862
[M-H]-	738.50293	5.77	PE 36:4|PE 18:2_18:2	0.3648±0.1712	0.1800±0.1575	9.9488±1.0807
[M-H]-	740.52386	6.245	PE 36:3|PE 18:1_18:2	3.0417±0.8821	1.8229±0.1208	8.5095±0.9396
[M-H]-	742.53058	6.408	PE 36:2|PE 18:1_18:1	0.2514±0.0641	0.1337±0.1229	0.7970±0.1462
[M-H]-	742.53131	7.137	PE 36:2|PE 18:0_18:2	16.1819±4.0786	3.6903±4.0228	5.1503±5.7086
[M-H]-	744.54767	7.709	PE 36:1|PE 18:0_18:1	5.7035±1.9239	3.2290±0.1271	3.0735±0.2357
[M-H]-	746.56238	6.314	PE 36:0|PE 18:0_18:0	0.0204±0.0047	0.0087±0.0085	0.0048±0.0011
[M-H]-	746.57208	8.342	PE 36:0|PE 17:0_19:0	0.2578±0.0672	0.0411±0.0018	0.0649±0.0418
[M-H]-	722.4563	6.555	PE 35:5|PE 15:1_20:4	0.2560±0.0607	0.0255±0.0036	0.0098±0.0032
[M-H]-	728.5127	6.741	PE 35:2|PE 17:0_18:2	0.0928±0.0175	0.2269±0.0516	0.2474±0.2794
[M-H]-	730.53033	7.322	PE 35:1|PE 17:0_18:1	0.1782±0.0558	0.4270±0.0384	0.4731±0.0597

[M-H]-	712.48828	5.763	PE 34:3|PE 16:0_18:3	0.0454±0.0216	0.1049±0.1140	1.4048±0.0682
[M-H]-	714.50153	6.366	PE 34:2|PE 16:0_18:2	0.8993±0.2538	1.4178±0.0602	12.1632±1.4442
[M-H]-	716.5257	6.773	PE 34:1|PE 16:0_18:1	0.7370±1.2749	3.0312±0.0767	5.2807±0.4092
[M-H]-	660.4743	5.436	PE 30:1|PE 16:0_14:1	0.0300±0.0043	0.5822±0.0605	0.4242±0.0730
[M-H]-	731.56201	8.001	PA 38:0|PA 19:0_19:0	0.1869±0.0454	0.0446±0.0039	0.0075±0.0040
[M-H]-	693.44867	3.582	PA 36:5|PA 18:2_18:3	0.0001±0.0002	0.0122±0.0008	0.6666±0.0887
[M-H]-	697.47882	4.812	PA 36:3|PA 18:1_18:2	0.0003±0.0003	0.0404±0.0398	1.9487±0.0591
[M-H]-	699.50189	5.461	PA 36:2|PA 18:1_18:1	0.0000±0.0000	0.0646±0.0660	0.4047±0.0197
[M-H]-	669.44885	4.139	PA 34:3|PA 16:0_18:3	0.0006±0.0005	0.0056±0.0041	0.2890±0.0322
[M-H]-	671.48962	5.609	PA 34:2|PA 16:0_18:2	0.0244±0.0366	2.4854±0.3502	0.2981±0.1676
[M-H]-	673.47852	5.467	PA 34:1|PA 16:0_18:1	0.0004±0.0007	0.0011±0.0015	0.6130±0.0016
[M-H]-	506.32236	2.255	LPE 20:1	0.3183±0.0992	0.0558±0.0055	0.0046±0.0025
[M-H]-	476.27463	1.349	LPE 18:2	1.1027±0.5833	4.2974±0.1677	0.7759±0.2499
[M-H]-	478.29214	1.697	LPE 18:1	1.6241±0.7821	7.2775±0.2920	0.5112±0.1649
[M-H]-	480.30713	2.306	LPE 18:0	7.6184±4.9658	3.4616±0.0149	0.1168±0.0360
[M-H]-	452.27655	1.647	LPE 16:0	0.8641±0.3632	6.4395±0.1764	0.4165±0.1525
[M+AcO-H]-	576.32971	1.464	LPC 18:3	0.0021±0.0015	0.9415±0.0524	0.0939±0.0242
[M+AcO-H]-	578.34662	1.749	LPC 18:2	0.3275±0.2573	8.0553±0.2602	1.7110±0.5060
[M+AcO-H]-	580.3559	2.238	LPC 18:1	0.0000±0.0000	3.8745±0.0374	0.7336±0.2349
[M+AcO-H]-	582.37347	3.029	LPC 18:0	1.3283±0.5557	2.5795±0.0570	0.1657±0.0044
[M+AcO-H]-	554.34692	2.187	LPC 16:0	0.1129±0.0467	8.1923±0.1886	0.9307±0.2398
[M+AcO-H]-	526.31287	1.563	LPC 14:0	0.1314±0.1593	0.3319±0.0285	0.0000±0.0000
[M-H]-	808.66168	7.827	HexCer 42:2;2O|HexCer 18:1;2O/24:1	0.6739±0.1718	0.0056±0.0004	0.0033±0.0022
[M-H]-	810.68158	8.372	HexCer 42:1;2O|HexCer 18:1;2O/24:0	0.9142±0.4121	0.0445±0.0023	0.0138±0.0002
[M+AcO-H]-	842.66949	7.858	HexCer 40:1;2O|HexCer 21:1;2O/19:0	0.2998±0.0893	0.4751±0.0697	0.2036±0.0747
[M+AcO-H]-	842.68085	7.769	HexCer 40:1;2O|HexCer 18:1;2O/22:0	0.2998±0.0893	0.4751±0.0697	0.2036±0.0747

[M-H]-	754.62671	7.278	HexCer 38:1;2O|HexCer 20:0;2O/18:1	0.1174±0.0200	0.0063±0.0008	0.0109±0.0028
[M-H]-	726.58801	6.654	HexCer 36:1;2O|HexCer 18:0;2O/18:1	0.0645±0.0135	0.0146±0.0017	0.0021±0.0011
[M-H]-	690.67242	8.537	Cer 45:1;2O|Cer 23:1;2O/22:0	0.0131±0.0041	0.0006±0.0008	0.0002±0.0000
[M+AcO-H]-	732.65466	8.546	Cer 44:3;2O|Cer 20:2;2O/24:1	0.0490±0.0254	0.0006±0.0009	0.0002±0.0002
[M+AcO-H]-	720.65027	8.706	Cer 43:2;2O|Cer 19:1;2O/24:1	0.1682±0.1073	0.0490±0.0016	0.0235±0.0065
[M+AcO-H]-	722.66241	9.199	Cer 43:1;2O|Cer 18:1;2O/25:0	0.1093±0.0561	0.1090±0.0170	0.0500±0.0132
[M-H]-	634.5	8.58	Cer 42:8;2O|Cer 20:2;2O/22:6	0.0333±0.0084	0.0011±0.0015	0.0002±0.0003
[M+AcO-H]-	706.63287	8.489	Cer 42:2;2O|Cer 18:1;2O/24:1	7.7252±3.2451	0.2334±0.0035	0.0439±0.0130
[M+AcO-H]-	708.65076	8.865	Cer 42:1;2O|Cer 18:1;2O/24:0	1.6624±1.3712	0.8080±0.0281	0.2179±0.0478
[M+AcO-H]-	710.66797	9.167	Cer 42:0;2O|Cer 18:0;2O/24:0	0.1485±0.1047	0.0782±0.0103	0.0429±0.0112
[M+AcO-H]-	692.61523	8.248	Cer 41:2;2O|Cer 17:1;2O/24:1	0.1368±0.0526	0.1436±0.1130	0.0379±0.0217
[M+AcO-H]-	694.63141	8.746	Cer 41:1;2O|Cer 18:1;2O/23:0	0.6277±0.2423	1.1061±0.0254	0.5096±0.1625
[M+AcO-H]-	696.66815	8.832	Cer 41:0;2O|Cer 18:0;2O/23:0	0.0978±0.0380	0.1967±0.0051	0.0540±0.0042
[M+AcO-H]-	666.53619	7.723	Cer 40:8;2O|Cer 18:2;2O/22:6	0.2376±0.0529	0.8712±0.0514	0.3365±0.1122
[M+AcO-H]-	678.59503	8.01	Cer 40:2;2O|Cer 18:2;2O/22:0	0.9085±0.3581	0.2044±0.0026	0.0173±0.0058
[M-H]-	618.57782	8.007	Cer 40:2;2O|Cer 16:1;2O/24:1	0.2056±0.0451	0.0005±0.0007	0.0001±0.0002
[M+AcO-H]-	680.62311	8.379	Cer 40:1;2O|Cer 18:1;2O/22:0	2.2338±0.4882	1.3354±0.0377	0.2600±0.1012
[M+AcO-H]-	682.63312	8.665	Cer 40:0;2O|Cer 16:0;2O/24:0	0.2303±0.0738	0.3795±0.0271	0.2011±0.0566
[M+AcO-H]-	666.60474	8.255	Cer 39:1;2O|Cer 17:1;2O/22:0	0.1041±0.0527	0.9416±0.0814	0.3270±0.1218
[M+AcO-H]-	666.60712	8.143	Cer 39:1;2O|Cer 16:1;2O/23:0	0.1041±0.0527	0.9416±0.0814	0.3270±0.1218
[M+AcO-H]-	668.61786	8.332	Cer 39:0;2O|Cer 16:0;2O/23:0	0.0444±0.0166	0.5676±0.0435	0.1320±0.0441
[M+AcO-H]-	652.58527	7.971	Cer 38:1;2O|Cer 18:1;2O/20:0	0.8649±0.2921	0.6219±0.0144	0.1180±0.0349
[M+AcO-H]-	652.58398	7.844	Cer 38:1;2O|Cer 16:1;2O/22:0	0.8649±0.2921	0.6341±0.0150	0.1243±0.0371
[M+AcO-H]-	654.58215	8.242	Cer 38:0;2O|Cer 16:0;2O/22:0	0.1556±0.0358	0.6094±0.0215	0.2047±0.0586
[M+AcO-H]-	622.52875	6.757	Cer 36:2;2O|Cer 18:2;2O/18:0	0.2230±0.1654	0.0342±0.0194	0.0076±0.0091
[M+AcO-H]-	624.5575	7.257	Cer 36:1;2O|Cer 18:1;2O/18:0	0.9276±0.4648	0.1532±0.0207	0.0468±0.0094

[M+AcO-H]-	626.59491	7.577	Cer 36:0;2O|Cer 18:0;2O/18:0	0.1448±0.0467	0.3052±0.0074	0.2403±0.0551
[M+AcO-H]-	596.52551	6.523	Cer 34:1;2O|Cer 18:1;2O/16:0	0.6786±0.5866	0.8799±0.0773	0.2484±0.0649
[M+AcO-H]-	582.51099	6.126	Cer 33:1;2O|Cer 17:1;2O/16:0	0.0364±0.0164	0.0818±0.0093	0.0207±0.0075
[M+AcO-H]-	568.49304	5.89	Cer 32:1;2O|Cer 18:1;2O/14:0	0.2625±0.0967	0.0960±0.0052	0.0401±0.0106
[M+AcO-H]-	568.49841	5.754	Cer 32:1;2O|Cer 16:1;2O/16:0	0.2625±0.0967	0.0960±0.0052	0.0401±0.0106
[M+AcO-H]-	570.50647	6.021	Cer 32:0;2O|Cer 16:0;2O/16:0	0.0256±0.0154	0.1151±0.0058	0.0286±0.0073
[M+AcO-H]-	512.4256	4.157	Cer 28:1;2O|Cer 18:1;2O/10:0	0.0778±0.0731	0.0008±0.0002	0.0008±0.0002
[M+AcO-H]-	514.45166	4.485	Cer 28:0;2O|Cer 18:0;2O/10:0	0.0174±0.0180	0.0010±0.0001	0.0004±0.0004

Table S4. Special lipid species detected in HM and absent from IF1

Adduct ion name	Average Mz	Average Rt(min)	Metabolite name
[M+NH4]+	922.78314	10.36	TG 56:7|TG 16:0_18:1_22:6
[M+NH4]+	840.70416	9.629	TG 50:6|TG 14:0_14:0_22:6
[M+H]+	813.68134	13.515	SM 42:2;2O|SM 18:1;2O/24:1
[M+H]+	815.69958	7.252	SM 42:1;2O|SM 18:1;2O/24:0
[M+H]+	787.60559	9.317	SM 41:8;2O|SM 20:3;2O/21:5
[M+H]+	776.55695	7.065	PE P-40:6|PE P-18:0_22:6
[M+H]+	778.57574	7.309	PE P-40:5|PE P-18:0_22:5
[M+H]+	780.58197	7.818	PE P-40:4|PE P-18:0_22:4
[M+H]+	748.5235	6.318	PE P-38:6|PE P-16:0_22:6
[M+H]+	750.54156	6.598	PE P-38:5|PE P-18:1_20:4
[M+H]+	752.55939	7.298	PE P-38:4|PE P-18:0_20:4
[M+H]+	752.5592	7.132	PE P-38:4|PE P-16:0_22:4
[M+H]+	724.52771	6.549	PE P-36:4|PE P-16:0_20:4
[M+H]+	726.54315	6.825	PE P-36:3|PE P-18:1_18:2
[M+H]+	728.55591	7.508	PE P-36:2|PE P-18:0_18:2
[M+H]+	700.52667	6.727	PE P-34:2|PE P-16:0_18:2
[M+H]+	792.54944	6.676	PE 40:6|PE 16:1_24:5
[M+H]+	796.60223	5.43	PC 37:4|PC 17:0_20:4
[M+H]+	810.60486	8.713	PC 38:4|PC 18:0_20:4
[M+H]+	788.60925	10.74	PC 36:1|PC 18:0_18:1
[M+H]+	762.59625	11.634	PC 34:0|PC 16:0_18:0
[M+NH4]+	528.51276	5.742	MG 29:1
[M+NH4]+	398.3233	2.057	MG 20:3

[M+NH4]+	528.40234	5.752	MG 30:8
[M+AcO-H]-	845.67133	3.383	SM 40:1;2O|SM 25:1;2O/15:0

Table S5. Special lipid species detected in HM and absent from IF2

Adduct ion name	Average Mz	Average Rt(min)	Metabolite name
[M+H]+	813.68134	13.515	SM 42:2;2O|SM 18:1;2O/24:1
[M+H]+	815.69958	7.252	SM 42:1;2O|SM 18:1;2O/24:0
[M+H]+	787.60559	9.317	SM 41:8;2O|SM 20:3;2O/21:5
[M+H]+	776.55695	7.065	PE P-40:6|PE P-18:0_22:6
[M+H]+	778.57574	7.309	PE P-40:5|PE P-18:0_22:5
[M+H]+	780.58197	7.818	PE P-40:4|PE P-18:0_22:4
[M+H]+	748.5235	6.318	PE P-38:6|PE P-16:0_22:6
[M+H]+	750.54156	6.598	PE P-38:5|PE P-18:1_20:4
[M+H]+	752.55939	7.298	PE P-38:4|PE P-18:0_20:4
[M+H]+	752.5592	7.132	PE P-38:4|PE P-16:0_22:4
[M+H]+	724.52771	6.549	PE P-36:4|PE P-16:0_20:4
[M+H]+	726.54315	6.825	PE P-36:3|PE P-18:1_18:2
[M+H]+	728.55591	7.508	PE P-36:2|PE P-18:0_18:2
[M+H]+	730.57214	8.08	PE P-36:1|PE P-16:0_20:1
[M+H]+	792.54944	6.676	PE 40:6|PE 16:1_24:5
[M+H]+	796.60223	5.43	PC 37:4|PC 17:0_20:4
[M+H]+	810.60486	8.713	PC 38:4|PC 18:0_20:4
[M+H]+	788.60925	10.74	PC 36:1|PC 18:0_18:1
[M+H]+	762.59625	11.634	PC 34:0|PC 16:0_18:0
[M+NH4]+	528.51276	5.742	MG 29:1
[M+AcO-H]-	871.68134	12.982	SM 42:2;2O|SM 18:1;2O/24:1
[M-H]-	792.54974	7.202	PE 40:5|PE 18:0_22:5

[M+AcO-H]-	526.31287	1.563	LPC 14:0

Table S6. Metabolic pathways of differential lipids

KEGG pathway	
map05417	Lipid and atherosclerosis
map05415	Diabetic cardiomyopathy
map05322	Systemic lupus erythematosus
map05231	Choline metabolism in cancer
map05212	Pancreatic cancer
map05200	Pathways in cancer
map05167	Kaposi sarcoma-associated herpesvirus infection
map05152	Tuberculosis
map05146	Amoebiasis
map05140	Leishmaniasis
map05132	Salmonella infection
map05130	Pathogenic Escherichia coli infection
map04979	Cholesterol metabolism
map04977	Vitamin digestion and absorption
map04975	Fat digestion and absorption
map04933	AGE-RAGE signaling pathway in diabetic complications
map04931	Insulin resistance
map04923	Regulation of lipolysis in adipocytes
map04920	Adipocytokine signaling pathway
map04912	GnRH signaling pathway
map04723	Retrograde endocannabinoid signaling
map04722	Neurotrophin signaling pathway
map04714	Thermogenesis
map04666	Fc gamma R-mediated phagocytosis

map04217	Necroptosis
map04140	Autophagy - animal
map04138	Autophagy - yeast
map04136	Autophagy - other
map04072	Phospholipase D signaling pathway
map04071	Sphingolipid signaling pathway
map04070	Phosphatidylinositol signaling system
map04024	cAMP signaling pathway
map01110	Biosynthesis of secondary metabolites
map01100	Metabolic pathways
map00603	Glycosphingolipid biosynthesis - globo and isoglobo series
map00600	Sphingolipid metabolism
map00592	alpha-Linolenic acid metabolism
map00591	Linoleic acid metabolism
map00590	Arachidonic acid metabolism
map00571	Lipoarabinomannan (LAM) biosynthesis
map00564	Glycerophospholipid metabolism
map00563	Glycosylphosphatidylinositol (GPI)-anchor biosynthesis
map00562	Inositol phosphate metabolism
map00561	Glycerolipid metabolism
map00260	Glycine, serine and threonine metabolism
